# Aggregation of Asian-American subgroups masks meaningful differences in health and health risks among Asian ethnicities: an electronic health record based cohort study

**DOI:** 10.1186/s12889-019-7683-3

**Published:** 2019-11-25

**Authors:** Nancy P. Gordon, Teresa Y. Lin, Jyoti Rau, Joan C. Lo

**Affiliations:** 1 Kaiser Permanente Division of Research, 2000 Broadway, Oakland, CA 94612 USA; 2 Kaiser Permanente Santa Clara Medical Center, 710 Lawrence Expressway, Santa Clara, CA 95051 USA

**Keywords:** Race/ethnicity, Health disparities, Prevalence, Asian American, Filipino, Chinese, South Asian, Japanese, Korean, Southeast Asian, Pacific Islander, Diabetes, Hypertension, Coronary artery disease, Obesity, Smoking, Cardiovascular risk factors, Population study

## Abstract

**Background:**

Few large cohort studies have examined the prevalence of diabetes mellitus (DM), hypertension (HTN), coronary artery disease (CAD), obesity, and smoking among middle-aged and older adults in the major Asian-American ethnic groups and Native Hawaiian/Pacific Islanders (PIs). The aim of this study was to evaluate how prevalence of these conditions and risk factors differs across Asian-American and PI ethnic groups and compares with an aggregated All Asian-American racial group.

**Methods:**

This study used a cohort of 1.4 million adults aged 45 to 84 who were Kaiser Permanente Northern California health plan members during 2016. The cohort included approximately 274,910 Asian-Americans (Chinese, Filipino, Japanese, Korean, Southeast Asian, South Asian, other), 8450 PIs, 795,080 non-Hispanic whites, 107,200 blacks, and 210,050 Latinos. We used electronic health record data to produce age-standardized prevalence estimates of DM, HTN, CAD, obesity (using standard and Asian thresholds), and smoking for men and women in all racial/ethnic subgroups and compared these subgroups to an aggregated All Asian-American racial group and to whites, blacks, and Latinos.

**Results:**

We found large differences in health burden across Asian-American ethnic subgroups. For both sexes, there were 16 and > 22 percentage point differences between the lowest and highest prevalence of DM and HTN, respectively. Obesity prevalence among Asian subgroups (based on an Asian BMI ≥ 27.5 kg/m^2^ threshold) ranged from 14 to 39% among women and 21 to 45% among men. Prevalence of smoking ranged from 1 to 4% among women and 5 to 14% among men. Across all conditions and risk factors, prevalence estimates for Asian-American and PI ethnic groups significantly differed from those for the All Asian-American group. In general, Filipinos and PIs had greater health burden than All Asians, with prevalence estimates approaching those of blacks.

**Conclusions:**

In a population of middle-aged and older adult Northern California health plan members, we found substantive differences in prevalence of chronic cardiovascular conditions, obesity, and smoking across Asian-American ethnic groups and between Asian-American ethnic groups and an aggregated All Asian racial group. Our study confirms that reporting statistics for an aggregated Asian-American racial group masks meaningful differences in Asian-American ethnic group health.

## Background

Asian-Americans are among the fastest growing of all major racial or ethnic groups in the United States [1]. Asians comprised 5% of the U.S. population in 2010, but are expected to be over 10% by 2050, a projected growth from 14 million to 38 million [2, 3]. However, there is scant information about Asian-American health and healthcare utilization. State and national health surveys have generally not reported statistics for Asian-Americans, in part due to survey subgroup samples that are insufficiently-sized for producing stable prevalence estimates. Furthermore, despite the cultural and sociodemographic heterogeneity of the Asian-American population, when Asian health data are reported, the statistics are seldom disaggregated by ethnic group and often include Native Hawaiian/Pacific Islanders (PI). Consequently, there is currently little available information to examine differences in health and healthcare use among Asian and PI ethnic groups and how health characteristics of individual Asian ethnic groups differ from characteristics of the broader Asian/PI group [4].

In 2011, as part of the U.S. Centers for Medicare and Medicaid Services (CMS) Stage 2 Meaningful Use (MU) requirements, health care organizations receiving CMS funds were incentivized to ascertain race/ethnicity and language preference directly from patients to populate their electronic health record (EHR) [5]. CMS intended for health care organizations to use this race/ethnicity information in conjunction with other EHR data to identify and reduce health and health care disparities [6]. To meet MU requirements, CMS deemed use of a single Asian race category as acceptable for recording and analyzing EHR racial/ethnicity data of people with different Asian ethnic backgrounds [6]. However, there is concern among researchers and advocacy groups in the medical and public health communities that the broad Asian or Asian/PI race category is too heterogeneous to be meaningful for research and reporting purposes because it may mask important differences across Asian ethnic groups [4, 7–10]. A 2009 report by the Institutes of Medicine (IOM) Subcommittee on Standardized Collection of Racial/Ethnicity Data for Healthcare Quality Improvement recommended that more granular race and ethnicity information be captured in EHRs [11].

Research comparing health status and health-related risks across multiple Asian-American ethnic groups is limited, but there is a growing body of evidence that health disparities exist by ethnicity within the broader Asian group. Studies conducted using national and state survey data have found significant differences across Asian ethnic groups in prevalence of obesity [12–15], smoking [12, 14, 16], diabetes [12, 17–19], high blood pressure [12, 15, 19], heart disease [12], and multiple chronic conditions [20]. However, despite use of pooled data from several survey cycles, the Asian ethnic subgroup samples used for these survey-based studies have been relatively small, leading to inconsistent findings across studies. Small subgroup samples have also limited the ability to examine variation across Asian ethnic groups separately for women and men and to focus analyses on the middle-aged and older adult segment of the population which has a significantly higher prevalence of chronic health conditions than the population aged 18 and over.

Mortality data and EHR-based studies have also shown differences among North American Asian ethnic groups. A Canadian death record study found that the mortality rate due to cardiovascular disease was significantly higher for South Asian than Chinese adults [21] and a U.S. study found higher CHD mortality rates among South Asians and Filipinos compared to Chinese, Japanese, Korean, and Vietnamese Americans [22]. The Canadian SHARE study based on clinical data for community-recruited samples of South Asian and Chinese adults aged 35–75 found that South Asians were more likely than Chinese adults to have a history of coronary heart disease, diabetes, and cardiovascular disease [23]. Studies conducted with medical record data for cohorts of Asian adult members of two Northern California health plans, Kaiser Permanente Northern California (KPNC) and Palo Alto Medical Foundation (PAMF), found significant variation across Asian ethnic groups in obesity [24], Type 2 diabetes [25, 26], hypertension [24], dyslipidemia [27], coronary heart disease [28, 29], stroke [29], peripheral vascular disease [29], and osteoporotic bone fractures [30].

In this study, we compared age-standardized prevalence of three cardiovascular conditions (diagnosed diabetes, hypertension, coronary artery disease), obesity, and smoking status among male and female KPNC health plan members aged 45–84 in six Asian-American (Chinese, Korean, Japanese, Southeast Asian, Filipino, and South Asian) and PI ethnic groups. We then compared these ethnic group prevalence estimates with prevalence for an aggregated Asian-American racial group and prevalence for whites, blacks, and Hispanic/Latinos. We focused on a middle and older adult age group because research on other populations suggests that the prevalence of these chronic conditions is very low among younger adults.

## Methods

### Setting

Kaiser Permanente Northern California (KPNC) provides integrated primary and specialty health care to a racial/ethnically and sociodemographically diverse membership that includes over 3.2 million adults who mostly reside in the San Francisco Bay and Greater Bay Area, Sacramento area, Silicon Valley, and Central Valley. The KPNC adult membership is very similar to the insured population of Northern California with regard to sociodemographic and health characteristics [31].

### Study population

To estimate and compare prevalence of health conditions, health risks, and health care utilization across Asian-American ethnic groups in KPNC and examine how reporting data for an aggregated Asian-American racial group may mask significant ethnic group variation, we created a race/ethnicity and language cohort, DECKA2016 (for Demographically Enriched Cohort of Kaiser Adults, calendar year 2016). This cohort is comprised of over 2.4 million adults aged 20–89 who were KPNC members for all 12 months of calendar year 2016 and for whom we could assign race/ethnicity based on information in their EHR, information from surveys and other data sources that was not entered into their EHR, and surname coding. Approximately half a million of the cohort members are Asians for whom we were able to assign to a specific Asian ethnic subgroup. A description of the methodology used to create our large population cohort and of the final cohort race/ethnicity subgroups is found in Additional file 1. We are able to link this cohort with EHR diagnosis, procedure, healthcare utilization, and Census-derived data to study variation in prevalence of chronic health conditions, obesity, smoking, and use of health care services across adult Asian-American ethnic groups. While this cohort was primarily created to study Asian ethnic groups, we also included data for white, black, and Hispanic/Latino adults for comparison purposes.

### Description of study cohort

For this study, we used DECKA2016 cohort data for 1.4 million members who were aged 45–84 on December 31, 2016 and had an EHR-coded sex of male or female. We restricted our Asian ethnic groups to adults we classified as Chinese (*n* = 87,128), Filipino (*n* = 88,691), Korean (*n* = 8910), Japanese (*n* = 16,886), Southeast Asian (*n* = 30,910), or South Asian (*n* = 35,565), but the aggregated All Asian group (*n* = 274,909) also includes Central/other Asian (*n* = 100, excluding Iranian/Persian), Chinese or Korean surname (*n* = 249), and known Asians whose ethnicity could not be specified based on available information or surname coding (Asian NSP, *n* = 6470). PIs (*n* = 8453), whites (*n* = 795,079), blacks (107,205), and Hispanic/Latinos (*n* = 210,050) were also included for comparison purposes. In this cohort, Chinese refers to people with ethnic origins in China, Taiwan, and Hong Kong, including ethnic Chinese living in other Asian countries. Southeast Asians were people not of Chinese ethnicity who had ethnic origins in Vietnam, Laos, Cambodia, Thailand, Indonesia, Singapore, Myanmar, or Malaysia. South Asians were those with ethnic origins in India, Pakistan, Afghanistan, Bangladesh, Sri Lanka, Nepal, or Bhutan, or who were Fijian Indian. Central/Other Asian does not include Iranian/Persian or Turkish, who are grouped with Middle Eastern. Native Hawaiian/Pacific Islanders included people whose race or ethnicity codes or primary language indicated Native Hawaiian, Fijian, Guamanian, Chamorro, Samoan, Polynesian, Tahitian, Tongan, or Micronesian. Hispanic/Latino adults were those identified as Hispanic/Latino race or ethnicity and met Office of Management and Budget (OMB) criteria for Latino (i.e., excludes people with ethnicity codes in the EHR for Spain, Italy, Portugal, Brazil, and other countries where Spanish was not the predominant language) [6].

Approximately 94% of PIs, 83% of Filipinos, 82% of Japanese, 81% of Chinese, 66% of Koreans, 60% of Southeast Asians, and 51% of South Asians were assigned to an ethnicity based on self-reported ethnicity information in their EHR or data from through clinical, research and operational patient questionnaires that did not feed into the EHR; the rest were assigned based on surname [32]. Of adults in the aggregated All Asian group, 17.7% had a preferred language other than English in the EHR (by ethnic group, 30.9% of Chinese, 31.7% of Korean, 32.1% of Southeast Asian, 10.8% of South Asian, 4.9% of Filipino, and 4.7% of Japanese), as did 2.0% of PIs. Approximately 3.4% of Asians were missing language preference data (range 1.6% of Filipinos to 5.1% of Chinese and 10% of Asian NSP). Table 1 shows the age distribution of the 11 racial/ethnic subgroups reported on in the subsequent tables.

**Table 1:** Age composition of study cohort ages 45–84 prior to standardization

Sex	Race/Ethnicity	45–54 yr		55–64 yr		65–74 yr		75–84 yr	
		N	%	N	%	N	%	N	%
All	All Asian	104,770	38.1	88,192	32.1	56,557	20.6	25,390	9.2
	Chinese	29,827	34.2	28,106	32.3	19,851	22.8	9344	10.7
	Korean	3235	36.3	2620	29.4	1993	22.4	1062	11.9
	Japanese	4428	26.2	5427	32.1	4342	25.7	2689	15.9
	Southeast Asian	15,307	49.5	9574	31.0	4545	14.7	1484	4.8
	Filipino	31,613	35.6	29,512	33.3	19,222	21.7	8344	9.4
	South Asian	17,029	47.9	10,701	30.1	5642	15.9	2193	6.2
	Native Hawaiian/Pacific Islander	3814	45.1	2882	34.1	1325	15.7	432	5.1
	White non-Hispanic	216,917	27.3	259,584	32.7	215,219	27.1	103,359	13.0
	African-American/Black	36,565	34.1	36,295	33.9	23,270	21.7	11,075	10.3
	Hispanic/Latino	91,848	43.7	65,763	31.3	34,753	16.6	17,686	8.4
Women	All Asian	56,397	37.9	47,591	32.0	30,917	20.8	14,000	9.4
	Chinese	16,559	35.2	15,129	32.2	10,616	22.6	4730	10.1
	Korean	1789	34.8	1553	30.2	1213	23.6	583	11.4
	Japanese	2650	26.8	2991	30.2	2476	25.0	1774	17.9
	Southeast Asian	7324	48.5	4734	31.3	2300	15.2	746	4.9
	Filipino	18,072	35.5	16,815	33.0	11,071	21.7	4999	9.8
	South Asian	7940	48.3	4898	29.8	2609	15.9	983	6.0
	Native Hawaiian/Pacific Islander	1839	45.4	1354	33.4	633	15.6	225	5.6
	White non-Hispanic	111,693	26.5	136,177	32.3	116,410	27.6	57,497	13.6
	African-American/Black	20,316	33.4	20,425	33.6	13,511	22.2	6544	10.8
	Hispanic/Latino	45,418	41.9	33,921	31.3	18,936	17.5	10,091	9.3
Men	All Asian	48,373	38.4	40,601	32.2	25,640	20.4	11,390	9.0
	Chinese	13,268	33.1	12,977	32.4	9235	23.0	4614	11.5
	Korean	1446	38.3	1067	28.3	780	20.7	479	12.7
	Japanese	1778	25.4	2436	34.8	1866	26.7	915	13.1
	Southeast Asian	7983	50.5	4840	30.6	2245	14.2	738	4.7
	Filipino	13,541	35.9	12,697	33.7	8151	21.6	3345	8.9
	South Asian	9089	47.5	5803	30.3	3033	15.9	1210	6.3
	Native Hawaiian/Pacific Islander	1975	44.9	1528	34.7	692	15.7	207	4.7
	White non-Hispanic	105,224	28.2	123,407	33.1	98,809	26.5	45,862	12.3
	African-American/Black	16,249	35.0	15,870	34.2	9759	21.0	4531	9.8
	Hispanic/Latino	46,430	45.7	31,842	31.3	15,817	15.6	7595	7.5

All Asian group includes aggregated data for the 6 Asian ethnic groups above plus other Asians not represented in the table (eg, Central Asians and those who could not be assigned to a more granular Asian ethnicity). This group does not include Native Hawaiian/Pacific Islanders

### Study variables

This study examined differences in prevalence of diabetes mellitus, hypertension, obesity, and current smoking for ages 45–84 and coronary artery disease (CAD), for ages 45–84 and 65–84 using EHR-derived data. The lists of ICD-9 and ICD-10 diagnosis codes for diabetes, hypertension, and CAD used to abstract EHR data for the cohort are found in Table 2**.** To be assigned as having a condition, adults must have had ≥1 office visit diagnosis code for that condition during calendar years 2015 and 2016 or a diagnosis code for that condition on the problem list in December 2016. Diabetes was assigned based on inclusion in the KPNC Diabetes Registry in December 2016, as identified through inpatient and outpatient diagnoses, lab test results, and pharmacy use (see Karter et al. for specifications [25]). BMI was calculated based on weight in EHR for the closest office visit to December 1, 2016 (date range: January 1, 2016 – December 31, 2016) and valid height on date closest to the date of that weight. For people with a suspected invalid height (< 40 in. or > 84 in.), weight (< 70 lbs. or > 500 lbs.) or BMI (< 14 or > 50), we examined additional heights and weights taken during that interval and either replaced the inaccurate values with a qualifying value or set BMI to missing. A valid BMI was available for approximately 74% of the All Asian group in our cohort aged 45–84 (range 72 to 76%). We classified Asians and other adults as obese based on the standard BMI threshold (≥ 30 kg/m^2^) and additionally classified Asians and PIs as obese using the lower Asian obesity threshold (≥ 27.5 kg/m^2^) recommended by the World Health Organization [33]. Smoking status (current smoker or non-smoker) was based on EHR tobacco use data on visit date closest to December 1, 2016. Adults who did not have smoking status data captured during calendar years 2015 or 2016 but who had information in their EHR for the three years prior (2012–2014) or three months after (January–March 2017) that indicated they had never smoked were coded as non-current smokers. Current smoking status was obtained for nearly 99% of all racial/ethnic groups.

**Table 2:** ICD codes used to assign cohort to chronic condition status

Condition	ICD-10 codes	ICD-9 codes
Diabetes Mellitus	Presence in KPNC Diabetes Registry (based on inpatient or outpatient ICD codes, lab test results and medication use. See Karter et al. article for specifics [25]	
Hypertension	I13.0, I13.1, I13.2, I13.9, I15.0, I15.8, I15.9, I10, I11.0, I11.9, I12.0, I12.9, H35.039	362.11, 401.0, 401.1, 401.9, 402.00, 402.01, 402.10, 402.11, 402.90, 402.91, 403.00, 403.01, 403.10, 403.11, 403.90, 403.91, 404.01, 404.03, 404.11, 404.13, 404.91, 404.93, 405.01, 405.09, 405.11, 405.19, 405.91, 405.99
Coronary artery disease	I20.0, I20.1, I20.8, I20.9, I21.02, I21.09, I21.11, I21.19, I21.21, I21.29, I21.3, I21.4, I24.0, I24.1, I25.19, I25.82, I25.810, I25.811, I25.812, I25.5, I25.89, I25.9	410.00, 410.01, 410.02, 410.10, 410.11,410.12, 410.20, 410.21, 410.22, 410.30, 410.31, 410.32, 410.40, 410.41, 410.42, 410.50, 410.51, 410.52, 410.60, 410.61, 410.62, 410.70, 410.71, 410.72, 410.80, 410.81, 410.82, 410.90, 410.91, 410.92, 411.0, 411.1, 411.81, 413.0, 413.1, 413.9, 414.02, 414.03, 414.04, 414.05, 414.06, 414.07, 414.2, 414.8, 414.9

### Data analysis

Study data were analyzed using SAS version 9.4 (SAS Institute, Cary, NC). To enable direct comparison of racial and ethnic groups unaffected by differences in the age-sex composition of the subgroups, we used a SAS Proc Surveyreg procedure recommended by the Centers for Disease Control and Prevention for direct standardization of prevalence estimates [34] using 2016 US Census data for men and women aged 45–84. Prevalence estimates for men and women were age-standardized using weighting factors from four age groups (45–54 years, 0.3367; 55–64 years, 0.3261; 65–74 years, 0.2252; 75–84 years, 0.1120). Prevalence estimates for men and women combined were age-sex standardized using eight age-sex subgroups (Male, Female x ages 45–54, 55–64, 65–74, 75–84). There were approximately equal percentages of men and women in each age group. Prevalence estimates restricted to ages 65–84 (CAD prevalence) were standardized using the distributions for two age groups (65–74 and 75–84). We compared non-standardized prevalence with the age- and age-sex standardized prevalence and found that they were nearly identical for almost every estimate, although standardized prevalence estimates for Japanese deviated from the non-standardized estimates by up to + 2 percentage points.

We created 99% confidence intervals around our prevalence estimates and used non-overlap of the confidence intervals of two racial/ethnic subgroups as a conservative indicator of a statistically significant difference at the *P* < .01 level. We defined meaningful differences between racial/ethnic subgroups (for men and women combined and by sex) and in comparison to the All Asian group as having non-overlapping 99% CIs and an absolute difference between the point estimates of at least 2 percentage points for prevalence estimates ≥ 10% or at least 1 percentage point for prevalence estimates that were both < 10%. Because our primary comparisons of interest were the Asian and PI ethnic groups with the aggregated All Asian group, we also calculated the absolute percentage point difference between each ethnic group and the All Asian group for men and women combined and within sex group. For comparison purposes, we provide the same statistics and analysis of meaningful differences from the All Asian subgroup for whites, blacks, and Hispanic/Latinos. We did not control for multiple comparisons. Because PIs are often combined with Asians in reporting of statistics, in our reporting of results, we refer to PIs in our low to high ranges of prevalence within the Asian group even though data for PIs were not included in the All Asian group estimates.

## Results

### Diabetes mellitus

Table 3 shows that the prevalence of diabetes for All Asians aged 45–84 was 23.1%, with a range of 15.6% for Chinese to 31.9% for Filipinos and 34.5% for PIs. There was a significant difference in prevalence by sex across all racial/ethnic groups. Diabetes prevalence for women in the All Asian group was 20.4% (range 13.4% of Chinese to 28.7% of Filipinas and 31.1% of PIs) and 26.1% for men (range 17.9% of Chinese to 35.4% of Filipinos and 38.3% of PIs). Prevalence estimates for East Asians (Chinese, Koreans, Japanese, and Southeast Asians) were ≥ 4 percentage points below that of the All Asian group, whereas prevalence estimates for Filipinos, and South Asians were ≥ 6 percentages points above and PI’s > 10 percentage points above the All Asian group.

**Table 3:** Standardized prevalence estimates of diabetes mellitus, ages 45–84, by race/ethnicity

	All 45–84 yr		Women 45–84 yr		Men 45–84 yr		All 45–84 yr		
		Age-sex standardized prevalence		Age-standardized prevalence		Age-standardized prevalence	Absolute percentage point difference from All Asian		
	N	% (99% CI)	N	% (99% CI)	N	% (99% CI)	All	Women	Men
All Asian	274,909	23.1% (22.9–23.3)	148,905	20.4% (20.1–20.7)	126,004	26.1% (25.8–26.5) ^b^	(ref) ^c^	(ref) ^c^	(ref) ^c^
Chinese	87,128	15.6% (15.2–15.9) ^a^	47,034	13.4% (13.0–13.8) ^a^	40,094	17.9% (17.4–18.4) ^a,b^	−7.5	−7.0	−8.2
Korean	8910	18.0% (17.0–19.0) ^a^	5138	14.8% (13.6–16.0) ^a^	3772	21.5% (19.8–23.2) ^a,b^	−5.1	−5.6	−4.6
Japanese	16,886	18.1% (17.4–18.9) ^a^	9891	14.5% (13.7–15.4) ^a^	6995	22.2% (20.9–23.4) ^a,b^	−4.9	−5.9	−3.9
Southeast Asian	30,910	18.7% (18.1–19.4) ^a^	15,104	16.8% (15.9–17.7) ^a^	15,806	21.0% (20.0–21.9) ^a,b^	−4.2	−3.6	− 5.1
Filipino	88,691	31.9% (31.5–32.3) ^a^	50,957	28.7% (28.2–29.2) ^a^	37,734	35.4% (34.8–36.0) ^a,b^	8.8	8.3	9.3
South Asian	35,565	29.1% (28.7–30.0) ^a^	16,430	25.5% (24.5–26.4) ^a^	19,135	33.7% (32.7–34.6) ^a,b^	6.3	5.1	7.6
Native Hawaiian/ Pacific Islander	8453	34.5% (33.1–35.9) ^a^	4051	31.1% (29.1–33.1) ^a^	4402	38.3% (36.3–40.3) ^a,b^	11.4	10.7	12.2
White non-Hispanic	795,079	12.8% (12.7–12.9) ^a^	421,777	10.6% (10.5–10.7) ^a^	373,302	15.3% (15.1–15.4) ^a,b^	−10.3	−10.2	−10.8
African-American/Black	107,205	24.9% (24.5–25.2)	60,796	23.3% (22.9–23.7) ^a^	46,409	26.6% (26.0–27.1) ^b^	1.8	2.9	0.5
Hispanic/ Latino	210,050	25.3% (25.0–25.5) ^a^	108,366	23.2% (22.8–23.5) ^a^	101,684	27.6% (27.3–28.0) ^b^	2.2	2.8	1.5

All Asian group includes aggregated data for the 6 Asian ethnic groups above plus other Asians not represented in the table. This group does not include Native Hawaiian/Pacific Islanders

^a^Non-overlapping 99% CIs and absolute percentage point difference from All Asian group within sex category of ≥ 2 percentage points

^b^Non-overlapping 99% CIs and ≥ 2 percentage point difference between women and men

^c^Ref: reference group for race and ethnic group comparisons

While diabetes prevalence for the All Asian group was similar to that of blacks and Latinos, diabetes prevalence for Filipinos, South Asians, and PIs was higher than that of blacks and Latinos, and diabetes prevalence for the East Asian groups was in between the white and black groups.

### Hypertension

Table 4 shows that the prevalence of diagnosed hypertension for All Asians aged 45–84 was 42.8%, with a range of 33.8% for Chinese to 56.1% for Filipinos and 53.1% for PIs. Significant sex differences were observed across most ethnic groups. Prevalence among women in the All Asian group was 41.6% (range 32.1% of Chinese to 55.6% of Filipinas) and 44.1% among men (range approximately 35.5% of Chinese and Koreans to 56.7% of Filipinos), with no significant sex difference observed for PIs. Hypertension prevalence for South Asians did not significantly differ from that of the All Asian group, whereas prevalence among Chinese, Koreans, and Southeast Asians was ≥ 7 percentage points below that of the All Asian group, and prevalence among Filipinos and PIs was > 11 percentage points higher than the All Asian group. Hypertension prevalence for Japanese was 3.2 percentage points lower than All Asian for men and women combined, but analysis by sex showed that prevalence was 6 percentage points lower for Japanese women, with no difference between All Asian and Japanese men.

**Table 4:** Standardized prevalence estimates of diagnosed hypertension, ages 45–84, by race/ethnicity

	All 45–84 yr		Women 45–84 yr		Men 45–84 yr		All 45–84 yr		
		Age-sex standardized prevalence		Age-standardized prevalence		Age-standardized prevalence	Absolute percentage point difference from All Asian		
	N	% (99% CI)	N	% (99% CI)	N	% (99% CI)	All	Women	Men
All Asian	274,909	42.8% (42.6–43.0)	148,905	41.6% (41.3–41.9)	126,004	44.1% (43.7–44.4) ^b^	(ref) ^c^	(ref) ^c^	(ref) ^c^
Chinese	87,128	33.8% (33.4–34.1) ^a^	47,034	32.1% (31.6–32.6) ^a^	40,094	35.6% (35.0–36.1) ^a,b^	−9.0	−9.5	−8.5
Korean	8910	33.9% (32.7–35.1) ^a^	5138	32.3% (30.8–33.8) ^a^	3772	35.5% (33.6–37.4) ^a^	− 8.9	− 8.3	−8.6
Japanese	16,886	39.6% (38.7–40.5) ^a^	9891	35.3% (34.1–36.4) ^a^	6995	44.3% (42.9–45.7) ^a,b^	−3.2	−6.3	0.2
Southeast Asian	30,910	35.7% (34.9–36.4) ^a^	15,104	34.2% (33.2–35.2) ^a^	15,806	37.2% (36.2–38.2) ^a,b^	−7.1	−7.4	− 6.9
Filipino	88,691	56.1% (55.7–56.5) ^a^	50,957	55.6% (55.1–56.1) ^a^	37,734	56.7% (56.1–57.3) ^a^	13.3	14.0	12.6
South Asian	35,565	43.4% (42.8–44.1)	16,430	41.5% (40.5–42.4)	19,135	45.5% (44.6–46.4) ^b^	0.6	−0.1	1.4
Native Hawaiian/ Pacific Islander	8453	53.1% (51.7–54.4) ^a^	4051	52.8% (50.8–54.7) ^a^	4402	53.4% (51.5–55.3) ^a^	11.3	11.2	9.3
White non-Hispanic	795,079	37.5% (37.4–37.7) ^a^	421,777	34.9% (34.9–35.0) ^a^	373,302	40.5% (40.3–40.6) ^a,b^	−5.3	−6.5	−3.6
African-American/Black	107,205	58.0% (57.6–58.3) ^a^	60,796	59.3% (58.9–59.8) ^a^	46,409	56.5% (55.9–57.0) ^a,b^	15.2	17.3	12.4
Hispanic/ Latino	210,050	42.4% (42.2–42.7)	108,366	41.8% (41.4–42.1)	101,684	43.1% (42.8–43.5)	−0.4	0.2	−1.0

All Asian group includes aggregated data for the 6 Asian ethnic groups above plus other Asians not represented in the table. This group does not include Native Hawaiian/Pacific Islanders

^a^Non-overlapping 99% CIs and absolute percentage point difference from All Asian group within sex category of ≥ 2 percentage points

^b^Non-overlapping 99% CIs and ≥ 2 percentage point difference between women and men

^c^Ref: reference group for race and ethnic group comparisons

Hypertension prevalence for the All Asian group was similar to that of Latinos, i.e., higher than whites and lower than blacks. Among women, hypertension prevalence among the East Asian groups was similar to that of whites, while prevalence for South Asians was similar to Latinas, and prevalence among Filipinos and PIs was closer to that of blacks. Among men, hypertension prevalence among Chinese, Koreans, and Southeast Asians was lower than that of whites, prevalence among Japanese and South Asians was similar to that of Latinos, and prevalence of Filipinos and PIs was similar to that of blacks.

### Coronary artery disease (CAD)

CAD prevalence was estimated for ages 45–84 and 65–84. In the text, we focus on prevalence among ages 65–84**.** Table 5 shows that the prevalence of CAD for All Asians aged 65–84 was 5.4%, with a range of 3.6% for Koreans to 8.3% for South Asians and 9.0% for PIs. A significant sex difference was observed across all ethnic groups. Prevalence among women in the All Asian group was 3.3% (range 1.7% for Koreans to approximately 4% for Filipinas and South Asians, and 5.9% for PIs), and 8.0% among men (range 5.6% of Southeast Asians to 13.0% of South Asians and 12.7% of PIs). CAD prevalence for Japanese and Southeast Asians was similar to that of the All Asian group, whereas prevalence among Chinese and Koreans was significantly lower and that of Filipinos, South Asians, and PIs was significantly higher. This same pattern was also seen in men aged 45–84 (see Additional file 2). CAD prevalence for the All Asian men group was 3.9, 6.4% for South Asian men, 7.4% for PI men, and 2.8% for Chinese and Korean men.

**Table 5:** Standardized prevalence estimates of diagnosed coronary artery disease, ages 65–84, by race/ethnicity

	All 65–84 yr		Women 65–84 yr		Men 65–84 yr		All 65–84 yr		
		Age-sex standardized prevalence		Age-standardized prevalence		Age-standardized prevalence	Absolute percentage point difference from All Asian		
	N	% (99% CI)	N	% (99% CI)	N	% (99% CI)	All	Women	Men
All Asian	81,947	5.4% (5.2–5.6)	44,917	3.3% (3.1–3.6)	37,030	8.0% (7.6–8.3) ^b^	(ref) ^c^	(ref) ^c^	(ref) ^c^
Chinese	29,195	4.2% (3.9–4.5) ^a^	15,346	2.5% (2.1–2.8)	13,849	6.4% (5.8–6.9) ^a,b^	−1.2	−0.8	−1.6
Korean	3055	3.6% (2.7–4.5) ^a^	1796	1.7% (0.9–2.5) ^a^	1259	5.9% (4.2–7.6) ^a,b^	−1.8	−1.6	−2.1
Japanese	7031	4.6% (3.9–5.3)	4250	2.7% (2.1–3.3)	2781	6.9% (5.7–8.1) ^b^	−0.8	−0.6	−1.1
Southeast Asian	6029	4.7% (3.9–5.4)	3046	3.8% (2.9–4.8)	2983	5.6% (4.5–6.8) ^a,b^	−0.7	0.5	−1.4
Filipino	27,566	6.5% (6.1–6.9) ^a^	16,070	4.3% (3.8–4.7)	11,496	9.2% (8.5–10.0) ^a,b^	1.1	1.0	1.2
South Asian	7835	8.3% (7.5–9.1) ^a^	3592	4.4% (3.5–5.3)	4243	13.0% (11.7–14.4) ^a,b^	2.9	1.1	5.0
Native Hawaiian/ Pacific Islander	1757	9.0% (7.2–10.8) ^a^	858	5.9% (3.8–8.0) ^a^	899	12.7% (9.7–15.7) ^a,b^	3.6	2.6	4.7
White non-Hispanic	318,578	5.9% (5.8–6.0)	173,907	3.7% (3.6–3.8)	144,671	8.5% (8.3–8.7) ^b^	0.5	0.4	0.5
African-American/Black	34,345	6.7% (6.4–7.1) ^a^	20,055	5.7% (5.3–6.1) ^a^	14,290	8.0% (7.4–8.5) ^b^	1.3	2.4	0.0
Hispanic/ Latino	52,439	6.0% (5.7–6.3)	29,027	4.3% (4.0–4.6)	23,412	8.1% (7.6–8.5) ^b^	0.6	1.0	0.1

All Asian group includes aggregated data for the 6 Asian ethnic groups above plus other Asians not represented in the table. This group does not include Native Hawaiian/Pacific Islanders

^a^Non-overlapping 99% CIs and absolute percentage point difference from All Asian group within sex category of ≥1 percentage point

^b^Non-overlapping 99% CIs and ≥ 1 percentage point difference between women and men

^c^Ref: reference group for race and ethnic group comparisons

CAD prevalence for the All Asian group was similar to that of whites. Among women, CAD prevalence among Chinese, Korean, and Japanese was slightly lower than that of whites, while prevalence for Southeast Asians was similar to whites, and prevalence for Filipinas and South Asians was not significantly different from whites and Latinas. Prevalence among PIs was similar to that of blacks, but because of the narrower age ranges, the CIs overlapped with both blacks and Latinas. Among men, Chinese, Korean, Japanese, and Southeast Asians had a lower CAD prevalence than whites, but CIs overlapped with those of blacks and Latinos. Filipinos had a slightly higher prevalence than whites, but with overlapping CIs, and prevalence among South Asians and PIs was significantly higher than whites, blacks and Latinos.

### Obesity

Tables 6 and 7**,** respectively, show differences in obesity using the standard BMI ≥ 30.0 kg/m^2^ threshold and lower ≥ 27.5 kg/m^2^ threshold recommended for Asians. Obesity prevalence in the All Asian group based on the standard threshold was 14.7%, ranging from 7.6 to 8.3% for Southeast Asians, Koreans, and Chinese to approximately 20% for South Asians and Filipinos and 44% for PIs. Obesity prevalence based on the lower Asian threshold was 29.5% for the All Asian group, ranging from approximately 19% for Chinese, Koreans, and Southeast Asians to 35 to 39% for Japanese, Filipinos, and South Asians and 61% for PIs. Based on the standard obesity threshold, with the exception of Southeast Asians, there were meaningful differences between women and men in obesity prevalence in the All Asian and individual Asian and PI ethnic groups. Using the lower Asian threshold, the differences between women and men were larger and significant for all Asian ethnic groups. For all Asian groups except South Asians, obesity prevalence was higher among men than women. PIs did not differ by sex using either obesity threshold. Using the standard obesity threshold, compared to the All Asian group, obesity prevalence was lower for Chinese, Koreans, and Southeast Asians, and higher for Japanese (men only), Filipinos, South Asians, and PIs (Table 6). Using the lower Asian obesity threshold **(**Table 7**)** increased the magnitude of the absolute differences between the All Asian and individual Asian ethnic groups.

**Table 6:** Standardized prevalence estimates of obesity based on BMI ≥ 30.0, ages 45–84, by race/ethnicity

	All 45–84 yr		Women 45–84 yr		Men 45–84 yr		All 45–84 yr		
		Age-sex standardized prevalence		Age-standardized prevalence		Age-standardized prevalence	Absolute percentage point difference from All Asian		
	N	% (99% CI)	N	% (99% CI)	N	% (99% CI)	All	Women	Men
All Asian	203,020	14.7% (14.5–14.9)	111,421	13.6% (13.3–13.9)	91,599	15.8% (15.5–16.1)^b^	(ref) ^c^	(ref) ^c^	(ref) ^c^
Chinese	62,912	8.3% (8.0–8.6) ^a^	34,576	6.8% (6.5–7.2) ^a^	28,336	9.9% (9.4–10.4) ^a,b^	−6.4	−6.4	−5.9
Korean	6510	7.6% (6.8–8.5) ^a^	3817	5.8% (4.8–6.8) ^a^	2693	9.6% (8.1–11.0) ^a,b^	−7.1	−7.4	− 6.2
Japanese	12,292	19.7% (18.8–20.7) ^a^	7257	15.5% (14.3–16.6)	5035	24.3% (22.7–26.0) ^a,b^	5.0	1.9	8.5
Southeast Asian	22,378	7.6% (7.1–8.0) ^a^	11,278	7.3% (6.6–7.9) ^a^	11,100	7.8% (7.2–8.5) ^a^	−7.1	−6.3	−8.0
Filipino	66,959	20.8% (20.4–21.2) ^a^	38,778	19.0% (18.5–19.5) ^a^	28,181	22.6% (22.0–23.3) ^a,b^	6.1	5.4	6.8
South Asian	27,113	20.0% (19.4–20.7) ^a^	12,581	22.4% (21.4–23.3) ^a^	14,532	17.4% (16.6–18.3) ^a,b^	5.3	8.8	1.6
Native Hawaiian/ Pacific Islander	5952	43.6% (41.9–45.4) ^a^	2885	44.5% (42.0–47.0) ^a^	3067	42.7% (40.3–45.0) ^a^	28.9	30.9	26.9
White non-Hispanic	576,700	36.4% (36.2–36.5) ^a^	304,556	34.7% (34.4–34.9) ^a^	272,144	38.2% (37.9–38.4) ^a,b^	21.7	21.1	22.4
African-American/Black	75,277	50.5% (50.0–50.9) ^a^	42,325	54.9% (54.2–55.5) ^a^	32,952	45.6% (44.9–46.3) ^a,b^	35.8	41.3	29.8
Hispanic/ Latino	154,675	44.6 (44.3–44.9) ^a^	81,307	44.5% (44.1–45.0) ^a^	73,368	44.6% (44.1–45.1) ^a^	29.9	30.9	28.8

All Asian group includes aggregated data for the 6 Asian ethnic groups above plus other Asians not represented in the table. This group does not include Native Hawaiian/Pacific Islanders

^a^Non-overlapping 99% CIs and absolute percentage point difference from All Asian group within sex category of ≥ 1 percentage point for percentages < 10% and ≥ 2 percentage points for percentages ≥ 10%

^b^Non-overlapping 99% CIs and ≥ 1 percentage point difference between women and men for percentages < 10% and ≥ 2 percentage points for percentages ≥10%

^c^Ref: reference group for race and ethnic group comparisons

**Table 7:** Standardized prevalence estimates of obesity based on BMI ≥30.0 for whites, blacks, and Hispanic/Latinos and BMI ≥27.5 for Asians and Pacific Islanders, ages 45–84, by race/ethnicity

	All 45–84 yr		Women 45–84 yr		Men 45–84 yr		All 45–84 yr		
		Age-sex standardized prevalence		Age-standardized prevalence		Age-standardized prevalence	Absolute percentage point difference from All Asian		
	N	% (99% CI)	N	% (99% CI)	N	% (99% CI)	All	Women	Men
All Asian	203,020	29.5% (29.2–29.8)	111,421	25.7% (25.3–26.0)	91,599	33.6% (33.2–4.0) ^b^	(ref) ^d^	(ref) ^d^	(ref) ^d^
Chinese	62,912	19.2% (18.8–19.6) ^b^	34,576	14.8% (14.3–15.3) ^a^	28,336	23.8% (23.2–24.5) ^a,b^	−10.3	−10.9	−9.8
Korean	6510	19.4% (18.1–20.6) ^b^	3817	13.6% (12.2–15.3) ^a^	2693	25.4% (23.3–27.6) ^a,b^	− 10.1	− 12.1	−8.2
Japanese	12,292	35.6% (34.5–36.8) ^b^	7257	26.8% (25.4–28.2)	5035	45.2% (43.3–47.0) ^a,b^	6.1	1.1	11.6
Southeast Asian	22,378	18.7% (18.0–19.4) ^b^	11,278	16.3% (15.4–17.3) ^a^	11,100	21.1% (20.1–22.2) ^a,b^	−10.8	−9.4	−12.5
Filipino	66,959	39.5% (39.0–40.0) ^b^	38,778	34.5% (33.9–35.2) ^a^	28,181	44.7% (44.0–45.5) ^a,b^	10.0	8.8	11.1
South Asian	27,113	38.2% (37.4–38.9) ^b^	12,581	39.3% (38.2–40.5) ^a^	14,532	36.8% (35.7–37.8) ^a,b^	8.7	13.6	3.2
Native Hawaiian/ Pacific Islander	5952	61.4% (59.7–63.1) ^b^	2885	60.6% (58.2–63.1) ^a^	3067	62.3% (59.9–64.6) ^a^	31.9	34.9	26.7
White non-Hispanic	576,700	36.4% (36.2–36.5) ^a^	304,556	34.7% (34.4–34.9) ^a^	272,144	38.2% (37.9–38.4) ^a,b^	6.9	9.0	4.6
African-American/Black	75,277	50.5% (50.0–50.9) ^a^	42,325	54.9% (54.2–55.5) ^a^	32,952	45.6% (44.9–46.3) ^a,b^	21.0	29.2	12.0
Hispanic/ Latino	154,675	44.6% (44.3–44.9) ^a^	81,307	44.5% (44.1–45.0) ^a^	73,368	44.6% (44.1–45.1) ^a^	15.1	18.8	11.0

All Asian group includes aggregated data for the 6 Asian ethnic groups above plus other Asians not represented in the table. This group does not include Native Hawaiian/Pacific Islanders

^a^Non-overlapping 99% CIs and absolute percentage point difference from All Asian group within sex category of ≥2 percentage points

^b^Non-overlapping 99% CIs and ≥ 2 percentage point difference between women and men

^c^Ref: reference group for race and ethnic group comparisons

Using the standard threshold for all racial/ethnic groups, prevalence of obesity was lower for the All Asian and individual Asian ethnic groups than for whites, blacks, and Latinos, with prevalence for PIs relatively close to that of whites. Using the lower threshold for Asians and PIs but retaining the standard threshold for whites, blacks, and Latinos decreased the absolute difference in obesity prevalence between the All Asian group and whites by approximately 10 percentage points for women and men combined and women only and by nearly 20 percentage points for men. Comparing obesity prevalence among the individual Asian ethnic groups to whites, blacks and Latinos using the lower threshold for Asians and PIs and higher for the other 3 groups, obesity prevalence for Japanese, Filipinos, and South Asians became similar to that for whites, Chinese, Korean, and Southeast Asian remained lower than that of whites, and prevalence for PIs was higher than that for blacks. Obesity prevalence of Japanese and Filipino men approximated that of blacks and Latinos, while prevalence among PI women and men was higher than that for blacks and Latinos.

### Smoking

Table 8 shows that the prevalence of smoking for All Asians aged 45–84 was 5.9%, with a range of 3.0% for South Asians to 7.7% for Koreans and 10.4% for PIs. However, there was a very large difference in smoking prevalence by sex across all racial/ethnic groups, with women having a much lower prevalence than men. Women in the All Asian group had a prevalence of 2.6% (range 0.9% for South Asians to 4.6% for Japanese and 7.8% for PIs), and prevalence among All Asian men was 9.5% (range 5.2% for South Asians to 13.8% for Southeast Asians and 13.3% for PIs). Compared to the All Asian women group, Chinese, Southeast Asian, and South Asian women were less likely to smoke, while Korean, Filipino, Japanese, and PI women were more likely to smoke. Compared to the All Asian men group, Chinese, Japanese, and South Asian men were less likely to smoke and Koreans, Southeast Asian, Filipino, and PI men were more likely to smoke.

**Table 8:** Standardized prevalence estimates of smoking, ages 45–84, by race/ethnicity

	All 45–84 yr		Women 45–84 yr		Men 45–84 yr		All 45–84 yr		
		Age-sex standardized prevalence		Age-standardized prevalence		Age-standardized prevalence	Absolute percentage point difference from All Asian		
	N	% (99% CI)	N	% (99% CI)	N	% (99% CI)	All	Women	Men
All Asian	262,809	5.9% (5.8–6.0)	144,439	2.6% (2.5–2.7)	118,370	9.5% (9.2–9.7) ^b^	(ref) ^c^	(ref) ^c^	(ref) ^c^
Chinese	82,396	4.8% (4.6–5.0)	45,358	1.4% (1.2–1.5) ^a^	37,038	8.4% (8.0–8.8) ^a,b^	−1.1	−1.2	−1.1
Korean	8370	7.7% (6.9–8.5) ^a^	4911	4.3% (3.5–5.0) ^a^	3459	11.4% (9.9–12.7) ^a.b^	1.8	1.7	1.9
Japanese	16,187	6.3% (5.8–6.8)	9601	4.6% (4.0–5.1) ^a^	6586	8.2% (7.3–9.0) ^a,b^	0.4	2.0	−1.3
Southeast Asian	28,798	7.3% (6.9–7.7) ^a^	14,460	1.3% (1.0–1.5) ^a^	14,338	13.8% (13.0–14.5) ^a,b^	1.4	−1.3	4.3
Filipino	86,274	7.3% (7.1–7.5) ^a^	49,913	4.0% (3.7–4.2) ^a^	36,361	10.9% (10.5–11.3) ^a,b^	1.4	1.4	1.4
South Asian	34,255	3.0% (2.7–3.2) ^a^	15,986	0.9% (0.7–1.2) ^a^	18,269	5.2% (4.7–5.6) ^a,b^	−2.9	−1.7	−4.3
Native Hawaiian/ Pacific Islander	8225	10.4% (9.6–11.3) ^a^	3963	7.8% (6.7–7.8) ^a^	4262	13.3% (11.9–14.6) ^a,b^	4.5	5.2	3.8
White non-Hispanic	772,492	8.6% (8.6–8.7) ^a^	412,414	7.7% (7.5–7.8) ^a^	360,078	9.7% (9.5–9.8) ^b^	2.7	5.1	0.2
African-American/Black	104,323	11.7% (11.4–11.9) ^a^	59,498	10.3% (9.7–10.6) ^a^	44,825	13.2% (12.8–13.6) ^a,b^	5.8	7.7	3.7
Hispanic/ Latino	203,154	6.4% (6.2–6.5)	106,218	4.5% (4.4–4.7) ^a^	96,936	8.3% (8.1–8.6) ^a,b^	0.5	1.9	−1.2

All Asian group includes aggregated data for the 6 Asian ethnic groups above plus other Asians not represented in the table. This group does not include Native Hawaiian/Pacific Islanders

^a^Non-overlapping 99% CIs and absolute percentage point difference from All Asian group within sex category of ≥ 1 percentage point

^b^Non-overlapping 99% CIs and ≥ 1 percentage point difference between women and men

^c^Ref: reference group for race and ethnic group comparisons

Compared to other race groups, smoking prevalence for All Asian women was below that of Latinas, while that for All Asian men was similar to that of whites. Among women, smoking prevalence among Koreans, Japanese, and Filipinas was similar to that of Latinas, while Chinese, Southeast Asians, and South Asians were less likely to smoke; PIs had a smoking prevalence similar to whites. Among men, South Asian smoking prevalence was lower than that of Latinos, was similar to that of whites for Chinese and Japanese men, slightly higher than whites for Filipino and Korean men, and similar to blacks for Southeast Asian and PI men.

### Summary of comparisons of Asian ethnic groups with the All Asian category

**Fig. 1:**
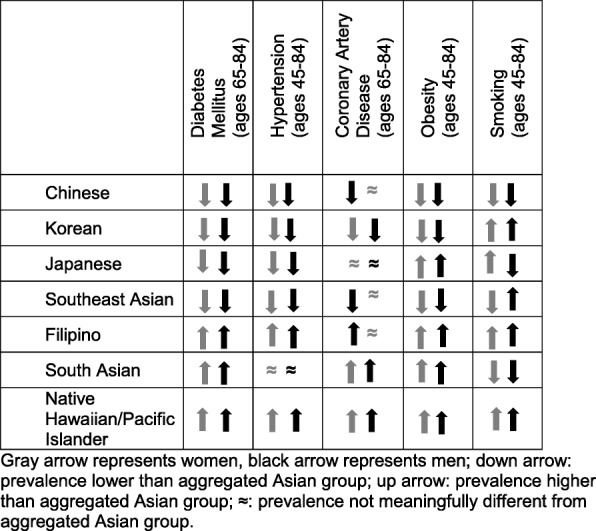
Summary of comparisons of Asian ethnic groups with aggregated Asian group on prevalence of chronic conditions, obesity, and smoking

Figure 1 summarizes comparisons of diabetes, hypertension, CAD, obesity and smoking prevalence estimates for women and men in the six Asian ethnic groups and PIs with those in the All Asian category. Across all conditions and risk factors examined, meaningful differences were observed for Filipino and PI men compared to All Asian men and women in these Asian ethnic groups, except for CAD prevalence among Filipina women which was similar to that of the All Asian women group. South Asian men had a higher prevalence of diabetes, CAD, and obesity, but were not meaningfully different from the All Asian group for hypertension, and they had a lower prevalence of smoking. South Asian women had a higher prevalence of diabetes, CAD, and obesity and lower smoking prevalence compared to All Asian women but were not meaningfully different for hypertension. Chinese and Korean men and women had lower prevalence of obesity and the chronic conditions than their counterparts among All Asians, with the exception that Chinese women were not meaningfully different from All Asian for CAD prevalence. However, in comparison to the All Asian group, Chinese men and women were less likely to be current smokers and Korean men and women were more likely to be smokers.

The comparisons for Japanese and Southeast Asians were mixed. Compared to All Asian, Japanese men and women had lower prevalence of diabetes, similar prevalence of CAD, a higher prevalence of obesity, and higher prevalence of smoking among women and lower among men. Southeast Asians exhibited more differences by sex than the other Asian ethnic groups. Compared to All Asian men, Southeast Asian men had lower prevalence of all the chronic conditions, a lower prevalence of obesity, and higher prevalence of smoking. Compared to All Asian women, Southeast Asian women similarly had a lower prevalence of diabetes, hypertension, and obesity, but were not meaningfully different from All Asian women regarding prevalence of CAD and were also less likely to be smokers.

## Discussion

In a 2012 article, Holland and Palaniappan summarized the limitations of most of the current data sources for studying Asian-American ethnic group differences in health and healthcare use [4]. They stressed the importance of collecting data for adequate sample sizes of Asian subgroups to produce stable prevalence estimates and enable comparisons among Asian ethnic groups, as well as including data collection for adults with limited English proficiency and low literacy who often choose not to participate in surveys and research. To our knowledge, ours is the largest cohort study to use EHR data to estimate prevalence of several chronic cardiovascular conditions (diabetes, hypertension, coronary artery disease) and cardiovascular risk factors (obesity and smoking) for an insured population of middle-aged and older Filipino, Chinese, Korean, Japanese, Southeast Asian, South Asian, and Pacific Islander adults in the U.S. and to compare prevalence statistics for these ethnic subgroups, overall and by sex, with those for an aggregated Asian group. Most previous studies of differences between U.S. Asian ethnic groups have estimated and compared prevalence of chronic cardiovascular conditions for populations that include younger adults, even though most of these chronic conditions are not diagnosed until middle or older age. By limiting our study population to adults aged 45–84 and producing separate age-standardized prevalence estimates for men and women, we believe that our study results provide a more precise comparison of statistics for the Asian ethnic groups with the aggregated Asian race group and with whites, blacks, and Latinos, the racial/ethnic groups that are the usual focus of racial/ethnic disparities studies.

Using data age-standardized to the U.S. Census population for ages 45–84, we found large variation across Asian ethnic groups in prevalence for all of the chronic conditions and risk factors we studied. For both men and women aged 45–84, the lowest and highest prevalence of diagnosed diabetes and hypertension differed by ≥ 15 percentage points and for obesity (using the Asian BMI threshold of BMI ≥ 27.5 kg/m^2^), the difference was > 20 percentage points. Using our criteria for meaningful differences (non-overlapping 99% CIs and absolute difference between prevalence estimates of ≥ 1 percentage point for comparison of prevalence estimates under 10% and ≥ 2 percentage points for prevalence estimates ≥ 10%), the only health condition for which the prevalence estimates for the majority of Asian ethnic groups were not meaningfully different from the aggregated Asian estimate was CAD in the 65–84 age group. However, the prevalence of CAD across all racial/ethnic groups was relatively low.

Overall, Filipinos, South Asians, and PIs tended to have meaningfully higher prevalence estimates than the aggregated Asian group for the chronic conditions, while East Asians (Chinese, Korean, Southeast Asian, and Japanese) had meaningfully lower prevalence estimates for these conditions. This pattern for East Asians did not extend to obesity prevalence, however, as both men and women in the Japanese group had higher prevalence of obesity than the aggregated Asian group, while Chinese, Koreans, and Southeast Asians had a lower prevalence. The pattern for ethnic group differences was also not observed for current smoking, where compared to the aggregated Asian group, prevalence was lower for South Asian men and women, higher for Korean men and women, lower for Japanese men and Southeast Asian women, and higher for Japanese women and Southeast Asian men.

Within racial/ethnic groups, our age-sex standardized prevalence estimates for men and women combined fell approximately midway between prevalence estimates for men and for women. Because of the very large numbers of women and men in our ethnic subgroups, we were able to show that the non-sex-specific age-sex standardized prevalence estimates for an Asian ethnic group in many cases over- or underestimated the age-standardized prevalence estimates for men and women in that ethnic group. In our study population, the age-sex standardized prevalence estimates for men and women combined did not substantively differ from age-standardized estimates for this group. However, this might not be true for populations where there is a greater sex imbalance within ethnic groups. Based on our findings, we recommend that when possible, prevalence of chronic conditions and risk factors should be estimated separately for women and men, and when this is not possible, standardized estimates should adjust for both age and sex.

Because we restricted our analyses to middle-aged and older adults rather than adults ages 18 and over (the population used for most national and state survey-based studies) or ages 35 and over (the population used for most of the PAMF cohort studies), the prevalence estimates from our study are not directly comparable to results of previously published studies. However, our finding that Filipinos and South Asians are at elevated risk for obesity, diabetes, hypertension, and CAD compared to other Asian ethnic groups is in line with results of several previous survey- and EHR-based studies [12, 24, 26, 29, 35, 36]. Additionally, our finding that estimated prevalence of these chronic conditions, obesity, and smoking for women and men in the aggregated Asian group significantly under- or overestimated prevalence for individual Asian ethnic subgroups confirms findings of other EHR- and mortality data-based studies [22, 24, 26, 29].

Our study results suggest that prevalence of chronic health conditions and cardiovascular risk factors in an ethnically diverse Asian population may be affected by the ethnic composition of the Asian group. For example, based on our findings, Asian populations with substantially larger proportions of Filipinos and South Asians than East Asian subgroups would be expected to have higher prevalence of diabetes, cardiovascular conditions, and obesity than Asian populations with larger proportions of East Asians. Forecasting healthcare service needs for a specific Asian population based on estimates for an All Asian group that does not have a similar Asian ethnic group composition to the one used for making the forecasts will potentially produce very inaccurate results. Additionally, comparing chronic disease prevalence, health risks, and healthcare quality metrics across different Asian populations without adjusting for differences in the Asian ethnic group composition of the populations may result in some geographic sub-regions or healthcare populations having a poorer health profile that is due to in part to the ethnic group differences. Also, we compared prevalence estimates for an All Asian/PI group versus All Asian group and found very little difference between the two groups despite the fact that PIs had higher prevalence of cardiovascular conditions and cardiovascular risks than the other Asian ethnic groups. However, this was because PIs were the second smallest group in the study population. The aggregated All Asian and All Asian/PI prevalence estimates grossly underestimated the actual prevalence estimates for the PI group.

Our results also suggest that information about Asian ethnicity in the EHR could lead to meaningful improvements in healthcare delivery, such as system-based prompts that help clinicians identify Asian patients in their adult or child panel who may be candidates for earlier screening and intervention and more frequent monitoring for diabetes and cardiovascular risk conditions. This may become important as care is increasingly delivered to patients through virtual (non-clinic based) encounters, limiting the visual and vital sign information that clinicians have available for monitoring and care planning. Information about Asian ethnicity can also help healthcare teams deliver more culturally competent care, such as using ethnically-tailored dietary assessments and providing culturally-tailored dietary advice and information resources when appropriate. At the population health management level, information about Asian ethnicity in the EHR would facilitate production of Asian-ethnic group specific quality metrics for chronic condition management, cancer screening, and immunizations that could be used to target and evaluate quality improvement efforts. Asian ethnicity information could also be used by health plans to build a medical facility workforce that mirrors the ethnic composition of the patient population being served.

While most health disparities research and policy has focused on the health and health risks of blacks and Hispanic/Latinos compared to whites, our study results suggest that Filipinos, South Asians, and PIs should also be considered higher risk groups compared to East Asians and as well as to whites. While the 2018 revised American Heart Association guidelines now identify South Asians as an ethnic group at heightened risk for atherosclerotic cardiovascular disease [37], no mention is made of heightened risk for Filipinos and PIs. In fact, across all of the cardiovascular conditions and risk factors we studied, prevalence among PIs was consistently higher than other Asian ethnic groups and the same or higher than that for blacks. We thus recommend that PIs never be grouped with Asians for estimating prevalence or as an adjusting factor in epidemiologic research, as this will mask the higher prevalence among PIs, and that information about an Asian/PI group not be extrapolated to forecast needs of a predominantly PI population.

Finally, our study results suggest that health plan member cohorts such as ours with very large Asian ethnic groups that can be linked to EHR data to study differences in health status, health risk factors, and healthcare utilization have great potential to inform clinical practice and public health policy and programs. However, collection of self-reported Asian ethnicity for entry into an EHR system using the ethnicity categories we used for this study or using more granular categories that can be aggregated into these ethnic subgroups would greatly facilitate future research and surveillance of Asian ethnic groups. At the data cleaning stage of the creation of our racial/ethnicity cohort, we found numerous people with a race of American Indian/Alaska Native in their EHR who had a primary language that was a South Asian Indian language or had a South Asian surname. This misclassification potentially could be avoided if there was an option to indicate South Asian (Indian, Pakistani, Afghani, etc.) in an expanded racial/ethnicity checklist when more granular ethnicity is not routinely collected. For U.S. patient populations, dropping “American” from granular ethnicity lists [11] might also improve ability to assign an Asian ethnicity based on EHR data.

Our study had several strengths. First, most previous studies of Asian ethnic group differences have relied on self-report health data obtained from surveys or study questionnaires, while we were able to categorize people based on ICD codes, measured heights and weights, and ascertainment of smoking at time of a clinic visit. Second, the very large numbers of men and women in each of our racial/ethnic subgroups enabled us to produce very stable prevalence estimates with very tight 99% confidence intervals. We were thus able to compare prevalence of the health characteristics for men and women within the same Asian ethnic group and to compare prevalence estimates for different Asian ethnic groups with estimates for All Asian group and other Asian ethnic groups separately by sex. The very large numbers in our racial/ethnic groups also enabled us to focus on middle-aged and older adults. This was important because prevalence of diagnosed diabetes and hypertension is very low before middle age and prevalence of coronary artery disease is very low even in middle age. Third, our study cohort included nearly all adults aged 20–89 who were health plan members during calendar year 2016, making it a truly representative population study. Study cohorts based on population surveys and people recruited for clinical research often under-represent adults who do not communicate well in English or have a very low level of education or literacy. Finally, because all of the adults in the study cohort were insured, receiving care from the same vertically integrated health care system, and living in the same geographic area, this reduced the potential for confounding due to healthcare access and geographic variability in health risk behaviors which is a limitation of studies based on national survey data.

We also acknowledge some potential limitations of the study. Some adults were assigned to an Asian ethnic group based on surname or first and last names, not self-reported or EHR-recorded data. However, we did not rely solely on automated (software-based) assignment of individuals by surname, but also compared first and last names of those assigned based on surname with Asian ethnicity codes for individuals assigned using the EHR and other self-reported data sources before finalizing assignments. Misclassification errors that may have occurred would likely have had limited impact on estimates of ethnic group prevalence statistics. In an EHR-based cohort study, Wang et al. found that across Asian ethnic groups, prevalence of diagnosed Type 2 diabetes among adults with self-identified race/ethnicity was similar to that for the full study cohort which included adults assigned to an Asian ethnicity based on surname [26]. Another potential source of racial/ethnic misclassification is that some individuals had more than one race or ethnicity. When this information came from the EHR, we used the algorithm described in the Methods section to assign individuals to one ethnicity category, but this may have resulted in inaccurate ethnic assignments for some people or confounding due to mixed ethnicity. We do not have information about how long these adults have lived in the U.S., nor what country they were born in if not in the U.S. Another potential limitation is that we did not restrict the cohort to adults who made at least one office visit in 2015 or 2016. This may have resulted in some adults with the chronic conditions we studied being missed, although we have no reason to suspect that under-identification due to non-utilization would be different across racial/ethnic groups. Based on KPNC clinical practice guidelines, middle-aged adults with diabetes, hypertension, and CAD should be routinely coming in to see a doctor or other healthcare provider at least annually and have a diagnosis refresh at time of receiving a medication refill. We further attempted to minimize missed diagnoses due to non-utilization by including diagnoses on the problem list during December 2016. There is always a possibility of inaccuracies in the EHR data, e.g., miscoded diagnoses or errors in data entry of height or weight information. Finally, while we consider the relative homogeneity of the study population with regard to geography and healthcare access as a strength, this may limit the generalizability of our results to uninsured and safety net populations or health plan populations in other geographic regions of the U.S.

## Conclusions

In a population of middle-aged and older adult Northern California Kaiser Permanente health plan members, we found meaningful differences between Asian-American ethnic groups in prevalence of chronic cardiovascular conditions and lifestyle risk factors. In most instances, the prevalence estimates for the All Asian group significantly differed from estimates for the individual Asian-American ethnic groups, confirming that reporting statistics for an aggregated Asian-American race group masks meaningful differences between Asian-American ethnic subgroups. We also found significant differences between men and women within racial/ethnic groups which were masked in the overall prevalence estimates for the ethnic groups. Our findings demonstrate the importance of disaggregating data for Asian ethnic groups and for men and women within ethnic groups in order to understand the burden of disease and risks of the heterogeneous Asian-American population and to apply this knowledge to the planning and delivery of healthcare.

## Supplementary information

**Additional file 1.** Description of the Kaiser Permanente Northern California 2016 Demographically Enriched Cohort of Kaiser Adults (DECKA2016)

**Additional file 2.** Table S5 b. Standardized prevalence estimates of diagnosed coronary artery disease, ages 45–84, by race/ethnicity

## Data Availability

The data supporting the results of this study are not publicly available due to privacy laws associated with medical data. Questions and requests regarding data availability should be addressed to the corresponding author (NPG).

## Abbreviations

BMI: Body mass index
CAD: Coronary artery disease
CMS: Centers for Medicare and Medicaid Services
DECKA2016: Demographically Enriched Cohort of Kaiser Adults 2016
DM: Diabetes mellitus
EHR: Electronic health record
HTN: Hypertension
ICD: International Classification of Diseases
IOM: Institutes of Medicine
KPNC: Kaiser Permanente Northern California
MU: Meaningful Use
PAMF: Palo Alto Medical Foundation
PI: Native Hawaiian/Pacific Islander
U.S.: United States of America
