# Supplementary material for: Aggregation of Asian-American subgroups masks meaningful differences in health and health risks among Asian ethnicities: an electronic health record based cohort study
Source: BMC Public Health. 2019 Nov 25;19:1551. doi: 10.1186/s12889-019-7683-3 (PMC6876105; doi:10.1186/s12889-019-7683-3)
Supplement: Supplementary file 1 — Additional file 1. Description of the Kaiser Permanente Northern California 2016 Demographically Enriched Cohort of Kaiser Adults (DECKA2016) [file 12889_2019_7683_MOESM1_ESM.pdf]

Description of the Kaiser Permanente Northern California 2016 Demographically Enriched Cohort of Kaiser Adults (DECKA2016)

Our first step in creating the Demographically Enriched Cohort of Kaiser Adults (DECKA2016) was to identify 2,567,714 adults who were between the ages of 20 and 89 as of December 31, 2016 and were members during all 12 months of calendar year 2016. We then used various sources of information to assign adults in the cohort to a single race/ethnicity. Specifically, we started with sources of self-reported race and ethnicity from operations and research surveys and clinical questionnaires. We next abstracted and coded race and ethnicity and preferred spoken and written language information available from Kaiser Permanente Northern California's electronic health record (EHR) and membership databases, and finally moved on to surname-based assignment (primarily to identify adults of South Asian and Arab ethnicity) [1]. Using this combination of sources, we were able to assign 96% (n=2,483,721) of adults to at least one race/ethnicity, restricting use of an "Other race" category to those who would be so classified using Office of Management and Budget (OMB) criteria [2]. Approximately 21% (531,608) of these adults were identified as at least partially Asian.

To assign known Asians with an unknown ethnicity to an Asian ethnic group, we first turned to information about their preferred spoken or written language. We then used vetted surname lists and crowd-sourced surname lists and websites to assign an ethnicity. Known Asians with Hispanic surnames who were not also coded as Hispanic in the EHR were presumed to be Filipino. Fijian Indians were coded as South Asians, and people whose race in the EHR was Native Hawaiian/Pacific Islander (PI) but whose ethnicity in the EHR suggested that they did not meet the OMB criteria for this category were recoded to a different racial or ethnic group or excluded from the cohort. Members with a South Asian surname or first name or who spoke a South Asian language were assigned as South Asians irrespective of how they were identified in the health plan records (e.g., some had either been identified as American Indian/Alaska Native, white, or Asian, or did not have an identified race in the EHR).

In situations where a name was common to more than one Asian ethnicity (a more common issue for East Asian and Southeast Asian surnames), assignment was made based on surname plus first name (possible with first names more common in certain countries); otherwise these people were classified as Chinese/Korean (if the name was common to both those ethnic groups but not Southeast Asian) or Asian non-specified (Asian NSP). Asians who could not be assigned to an ethnic group based on surname or language preference were also kept as Asian NSP. Because of ethnic diaspora, some Asians ultimately coded as Chinese

might actually have familial ties to Southeast Asian (e.g., Singapore or Malaysia) or the Philippines, and some adults coded as Japanese (based on Asian race and surname) might have familial ties to Brazil or Mexico. We also used surname lists and language preference information to separate adults with Middle Eastern/North African and Iranian surnames from the white, black, and South Asian subgroups. Much of the surname-based race/ethnicity assignment, including data cleaning, was done manually by the lead author. In cases where a member had more than one non-overlapping race and/or ethnicity (e.g., overlapping categories might include Asian NSP and a specific Asian ethnicity), we prioritized assignment to one race/ethnicity as follows: Hispanic/Latino, followed by African-American/black, Asian (and priority within Asian, to Chinese, Filipino, Southeast Asian, Japanese, Korean, South Asian, Chinese or Korean, Central Asian excluding Iranian/Persian, Asian NSP), PI, American Indian/Native American (AI/AN), Middle Eastern/North African (excluding Israeli, Iranian/Persian, and Turkish), and finally white (no other race or ethnicity indicated).

The final DECKA2016 cohort is comprised of 1,237,238 non-Hispanic whites, 188,057 African-American/Blacks, 451,217 Hispanics, 531,611 Asian, 36,720 Middle Eastern (including Iranian-Persian and Turkish), and 17,736 Other. By ethnicity, the Asian group includes 152,569 Filipinos, 149,418 Chinese, 15,990 Korean, 720 Chinese or Korean, 74,043 Southeast Asian, 96,803 South Asian, 25,060 Japanese, and 348 Central and other Asian (excluding Iranian/Persian), and 16,660 who could not be assigned to an Asian ethnicity (see Table A1 for counts by age group overall and by sex). In this cohort, Chinese refers to people with ethnic origins in China, Taiwan, and Hong Kong, including ethnic Chinese living in other Asian countries. Southeast Asians were people not of Chinese ethnicity who had ethnic origins in Vietnam, Laos, Cambodia, Thailand, Indonesia, Singapore, Myanmar, or Malaysia. South Asians were those with ethnic origins in India, Pakistan, Afghanistan, Bangladesh, Sri Lanka, Nepal, or Bhutan, or who were Fijian Indian. Central/Other Asian does not include Iranian/Persian or Turkish, who were grouped with Middle Eastern/North African. Native Hawaiian/Pacific Islanders included people whose race or ethnicity codes or primary language indicated Native Hawaiian, Fijian, Guamanian, Chamorro, Samoan, Polynesian, Tahitian, Tongan, or Micronesian. Hispanic/Latino adults were those identified as Hispanic/Latino race or ethnicity and met OMB criteria for Latino (i.e., excludes people with ethnicity codes in the EHR for Spain, Italy, Portugal, or countries were Spanish was not the predominant language).

Table A1. Age composition of DECKA2016 study cohort ages 20-89

**Race/ethnicity**				**Age**			
	**20-34 yr**	**35-44 yr**	**45-54 yr**	**55-64 yr**	**65-74 yr**	**75-89 yr**	**20-89 yr**
White NH	234,500	176,986	216,917	259,584	215,219	131,289	123,4495
Black	45,511	30,619	36,565	36,295	23,270	13,503	185,763
Hispanic	136,804	99,888	91,848	65,763	34,753	22,165	451,221
Asian	139,827	118,313	104,770	88,192	56,557	31,177	538,836
Chinese	35,432	28,534	29,827	28,106	19,851	11,787	153,537
Korean	3,244	3,783	3,235	2,620	1,993	1,212	16,087
Japanese	3,864	3,579	4,428	5,427	4,342	3,727	25,367
Southeast Asian	22,118	19,210	15,307	9,574	4,545	1,708	72,462
Filipino	34,997	30,362	31,613	29,512	19,222	9,904	155,610
South Asian	33,498	28,733	17,029	10,701	5,642	2,525	98,128
Other Asian or Asian NSP[a]	6,674	4,112	3,331	2,252	962	314	17,645
Pacific Islander	5,893	4,371	3,814	2,882	1,325	490	18,775
Middle East/Arab North African	9,420	7,252	6,807	6,653	4,099	2,493	36,724
Other	2,741	2,166	2,830	3,891	3,592	2,687	17,917
Total	574,696	439,595	463,551	463,260	338,815	203,804	248,3721

[a] Asian NSP: Asians who could not be assigned to an ethnic group; Other Asian includes Central Asian republics and Mongolia.

Approximately 77% of Filipinos and Japanese, 70% of Chinese, 56% of Koreans, 51% of Southeast Asians, 44% of South Asians, and 95% of Native Hawaiian/Pacific Islanders in the full study cohort (ages 20-89) were assigned to an ethnicity based on EHR data or race/ethnicity data available through clinical, research and operational data sources that did not feed into the EHR, with the rest assigned based on surname. Table A2 provides a breakdown of the percentages of each Asian ethnic subgroup assigned based on EHR or self-reported ethnicity data for ages 20-44, 45-64, and 65-89.

Table A2. Percentages of the DECKA2016 cohort aged 20-89 who were assigned to an Asian or Pacific Islander ethnic group based on race/ethnicity data from electronic health records[a] or self-reported questionnaires

	20-89 yr	20-44 yr	45-64 yr	65-89 yr
Chinese	70.2%	55.0%	75.3%	91.5%
Korean	56.6%	43.7%	59.6%	79.6%
Japanese	76.8%	62.7%	75.5%	91.3%
Southeast Asian	50.3%	42.8%	56.9%	73.7%
Filipino	77.6%	69.6%	79.6%	91.7%
South Asian	44.3%	40.3%	46.4%	67.2%
Native Hawaiian/ Pacific Islander	94.2%	94.1%	94.4%	95.4%

[a] Includes primary language

References

1. Wong EC, Palaniappan LP, Lauderdale DS. Using name lists to infer Asian racial/ethnic subgroups in the healthcare setting. Med Care. 2010; 48(6): 540–546. doi:10.1097/MLR.0b013e3181d559e9.

2. Office of Management and Budget. Revisions to the Standards for the Classification of Federal Data on Race and Ethnicity. Federal Register / Vol. 62, No. 210 / Thursday, October 30, 1997. Available at: https://www.govinfo.gov/content/pkg/FR-1997-10-30/pdf/97-28653.pdf. [Accessed 20 Sept 2019]
