# Supplementary material for: Aggregation of Asian-American subgroups masks meaningful differences in health and health risks among Asian ethnicities: an electronic health record based cohort study
Source: BMC Public Health. 2019 Nov 25;19:1551. doi: 10.1186/s12889-019-7683-3 (PMC6876105; doi:10.1186/s12889-019-7683-3)
Supplement: Supplementary file 2 — Additional file 2. Table S5 b. Standardized prevalence estimates of diagnosed coronary artery disease, ages 45–84, by race/ethnicity [file 12889_2019_7683_MOESM2_ESM.docx]

| Table 5b. Standardized prevalence estimates of diagnosed coronary artery disease, ages 45-84, by race/ethnicity | | | | | | | | | | | |
| --- | --- | --- | --- | --- | --- | --- | --- | --- | --- | --- | --- |
|  | **All 45-84 yr** | | **Women 45-84 yr** | | | **Men 45-84 yr** | | **All 45-84 yr** | | | |
|  |  | **Age-sex standardized prevalence** |  | | **Age-standardized prevalence** |  | **Age-standardized prevalence** | **Absolute percentage point difference from All Asian** | | | |
|  | N | **% (99% CI)** | | N | **% (99% CI)** | N | **% (99% CI)** | **All** | | **Women** | **Men** |
| **All Asian** | **274,909** | **2.6% (2.5-2.7)** | | **148,905** | **1.5% (1.4-1.6)** | **126,004** | **3.9% (3.8-4.1)** ^b^ | **(ref)** ^c^ | **(ref)** ^c^ | | **(ref)** ^c^ |
| Chinese | 87,128 | 1.8% (1.7-1.9) | | 47,034 | 1.0% (0.9-1.1) | 40,094 | 2.8% (2.6-3.0) ^a,b^ | -0.8 | -0.5 | | -1.1 |
| Korean | 8,910 | 1.7% (1.3-2.0) | | 5,138 | 0.7% (0.4-1.0) | 3,772 | 2.8% (2.1-3.5) ^a,b^ | -0.9 | -0.8 | | -1.1 |
| Japanese | 16,886 | 2.1% (1.9-2.4) | | 9,891 | 1.2% (0.9-1.5) | 6,995 | 3.2% (2.7-3.7) ^b^ | -0.5 | 0.3 | | -0.7 |
| Southeast Asian | 30,910 | 2.1% (1.9-2.4) | | 15,104 | 1.5% (1.2-1.9) | 15,806 | 2.8% (2.4-3.3) ^b^ | -0.4 | 0.0 | | -1.1 |
| Filipino | 88,691 | 3.3% (3.1-3.4) | | 50,957 | 1.9% (1.8-2.1) | 37,734 | 4.8% (4.5-5.1) ^b^ | 0.7 | 0.4 | | 0.9 |
| South Asian | 35,565 | 4.2% (3.9-4.5) ^a^ | | 16,430 | 2.1% (1.7-2.4) | 19,135 | 6.6% (6.0-7.1) ^a,b^ | 1.6 | 0.6 | | 2.7 |
| Native Hawaiian/  Pacific Islander | 8,453 | 5.1% (4.4-5.8) ^a^ | | 4,051 | 3.0% (2.2-3.9) ^a^ | 4,402 | 7.4% (6.2-8.5) ^a,b^ | 2.5 | 1.5 | | 3.5 |
| White  non-Hispanic | 795,079 | 2.8% (2.7-2.8) | | 421,777 | 1.7% (1.6-1.7) | 373,302 | 4.0% (3.9-4.1) ^b^ | 0.2 | 0.2 | | 0.1 |
| African-American/Black | 107,205 | 3.4% (3.2-3.5) | | 60,796 | 2.8% (2.6-3.0) | 46,409 | 4.0% (3.8-4.3) ^b^ | 0.8 | 1.3 | | 0.1 |
| Hispanic/  Latino | 210,050 | 2.8% (2.7-2.9) | | 108,366 | 1.9% (1.8-2.0) | 101,684 | 3.8% (3.6-3.9) ^b^ | 0.2 | 0.4 | | -0.1 |
| All Asian group includes aggregated data for the 6 Asian ethnic groups above plus other Asians not represented in the table. This group does not include Native Hawaiian/Pacific Islanders.  ^a^ Non-overlapping 99% CIs and absolute percentage point difference from All Asian group within sex category of ≥ 1 percentage point.  ^b^ Non-overlapping 99% CIs and ≥1 percentage point difference between women and men.  ^c^ Ref: reference group for race and ethnic group comparisons | | | | | | | | | | | |
